# Binding of a fluorescence reporter and a ligand to an odorant-binding protein of the yellow fever mosquito, *Aedes aegypti*

**DOI:** 10.12688/f1000research.5879.2

**Published:** 2015-01-09

**Authors:** Gabriel M. Leal, Walter S. Leal

**Affiliations:** 1 Department of Molecular and Cellular Biology, University of California, Davis, Davis, CA, 95616, USA; 2 Davis Senior High School, Davis, CA, 95616, USA

## Abstract

Odorant-binding proteins (OBPs), also named pheromone-binding proteins when the odorant is a pheromone, are essential for insect olfaction. They solubilize odorants that reach the port of entry of the olfactory system, the pore tubules in antennae and other olfactory appendages. Then, OBPs transport these hydrophobic compounds through an aqueous sensillar lymph to receptors embedded on dendritic membranes of olfactory receptor neurons. Structures of OBPs from mosquito species have shed new light on the mechanism of transport, although there is considerable debate on how they deliver odorant to receptors. An OBP from the southern house mosquito, *Culex quinquefasciatus,* binds the hydrophobic moiety of a mosquito oviposition pheromone (MOP) on the edge of its binding cavity. Likewise, it has been demonstrated that the orthologous protein from the malaria mosquito binds the insect repellent DEET on a similar edge of its binding pocket. A high school research project was aimed at testing whether the orthologous protein from the yellow fever mosquito, AaegOBP1, binds DEET and other insect repellents, and MOP was used as a positive control. Binding assays using the fluorescence reporter N-phenyl-1-naphtylamine (NPN) were inconclusive. However, titration of NPN fluorescence emission in AaegOBP1 solution with MOP led to unexpected and intriguing results. Quenching was observed in the initial phase of titration, but addition of higher doses of MOP led to a stepwise increase in fluorescence emission coupled with a blue shift, which can be explained at least in part by formation of MOP micelles to house stray NPN molecules.

## Introduction

Over the past decade progress towards our understanding of the molecular basis of mosquito olfaction has been remarkable. It was not until the sunset of last century that odorant receptor (OR) genes have been identified in the genome of the fruit fly, *Drosophila melanogaster* ^1– 3^ and thereafter in mosquitoes and various insect species (see review ^4^), and less than a decade since the unique topology of ORs, with an intracellular N-terminus and an extracellular C-terminus ^5^, has been elucidated. Although previously known from moth species ^6^, it was about a decade ago that the first odorant-binding proteins (OBPs) from mosquitoes have been isolated and identified ^7^. By now the complete repertoire of olfactory genes, including *OBP, OR* and *ionotropic receptor* ( *IR*) genes, have been identified in the three major mosquito species: the yellow fever mosquito, *Aedes aegypti* ^8^, the malaria mosquito, *Anopheles gambiae* ^9^, and the southern house mosquito, *Culex quinquefasciatus* ^10^. There is growing evidence in the literature that OBPs and ORs play a crucial role in the sensitivity and selectivity of the insect’s olfactory system ^4^. Mosquito ORs have been deorphanized and demonstrated to be essential for the reception of physiologically and behaviorally relevant odorants ^9, 11^, including oviposition attractants ^12– 14^, insect repellents ^15^ and a signature compound (sulcatone) for human host preference ^16^. Elucidation of the three-dimensional (3D) structures of mosquito OBPs ^17– 21^ along with knockdown experiments ^22, 23^ and binding assays ^24– 27^ strongly suggest that these olfactory proteins are involved in the transport of odorant from the ports of entry of olfactory sensilla (the pore tubules) to ORs housed on dendritic membranes of olfactory receptor neurons.

**Figure 1.**
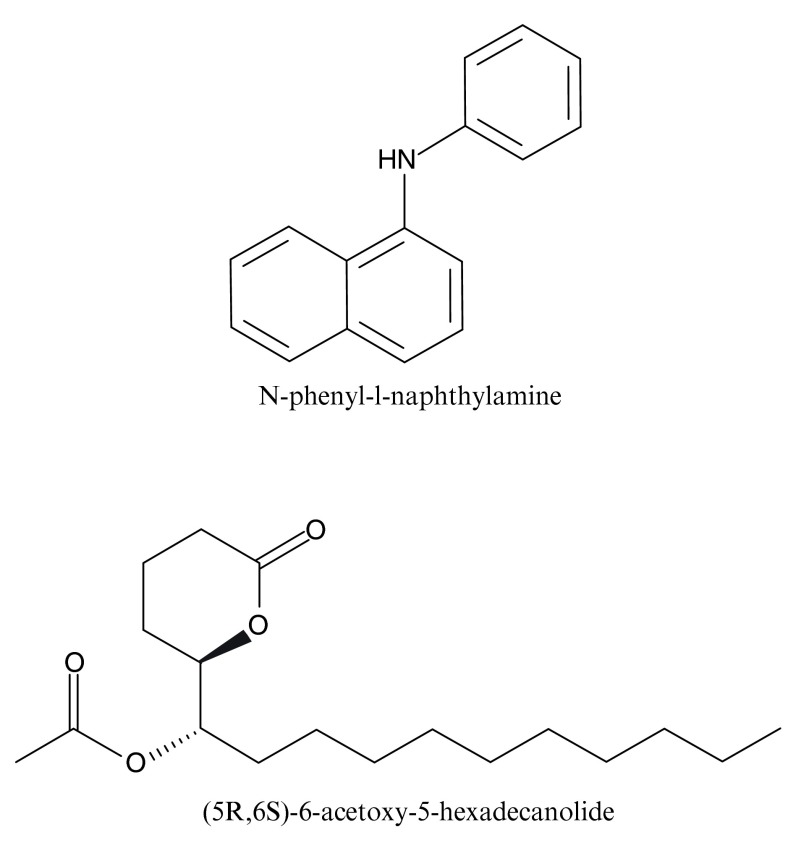
Structures of a fluorescence reporter and a mosquito oviposition pheromone. N-phenyl-1-naphthylamine (NPN) is widely used in binding assays with insect OBPs. (5 *R*,6 *S*)-6-acetoxy-5-hexadecanolide (MOP) is an attractant first isolated from eggs of *Cx. quinquefasciatus* ^37^, but it is known to bind not only to CquiOBP1, but also to its orthologous proteins, i.e., AaegOBP1 and AgamOBP1 ^19^.

There are typically two binding assays to “de-orphanize” OBPs, i.e., to measure their binding affinities and specificity towards physiologically and behaviorally relevant odorants (ligands). They are the cold binding assay ^28^ so named because – as opposed to its predecessors - it does not require radioactive ligands and a fluorescence reporter assay ^29, 30^. The former is based on separation of bound and unbound OBPs, followed by extraction of bound ligands and their quantification by gas chromatography. In the latter a test OBP is bound to a fluorescence reporter, *N*-phenyl-1-naphthylamine (NPN, Figure 1), and subsequently increasing amounts of a test ligand are added. Decreasing NPN fluorescence emission is inferred as NPN displacement, i.e., the test ligand is assumed to compete for the binding site initially occupied by NPN. The fluorescence reporter assay is such a facile method that we envisioned it could be used even in a high school research project.

The 3D structures of the malaria mosquito OBP, AgamOBP1 ^21^ bound to polyethylene glycol (PEG) and AgamOBP1 complex with DEET ^18^, suggested that AgamOBP1 could be a DEET carrier. For this high school project we asked the question whether DEET and other insect repellents (picaridin, IR3535, and PMD) would bind to AaegOBP1 ^31^ (also named AaegOBP39 ^32, 33^), an orthologue of AgamOBP1 from the yellow fever mosquito with similar 3D structure ^20^. In the course of this investigation, we found evidence suggesting that AaegOBP1 might bind simultaneously the fluorescence reporter and an odorant.

## Materials and methods

### Protein preparations

AaegOBP1 (AY189223) ^31^ was expressed in LB medium with transformed BL21(DE3) cell (Agilent Technologies, Santa Clara, CA) according to a protocol for periplasmic expression of insect OBPs ^34^. Proteins were extracted with 10 mM Tris-HCl, pH 8 by three cycles of freeze and thaw ^35^. After centrifuging at 16,000×g to remove debris, AaegOBP1 was isolated from the supernatant and purified by a series of ion-exchange and gel filtration chromatographic steps, as previously described ^20^. The purest fractions were combined and desalted, according to a previous protocol ^20^. Then, AaegOBP1 was delipidated following an earlier protocol ^36^ with small modifications. In short, hydroxyalkoxypropyl-dextran Type VI resin (H2658, Sigma, St. Louis, MI) (1g) was suspended in HPLC grade methanol (20 ml), transferred to a glass column (i.d., 8.5 mm) with a stopper, washed with 60 ml of methanol and then washed and finally equilibrated with 50 mM citric acid buffer, pH 4.5. AaegOBP1 (ca. 2 mg per batch) in 50 mM citric acid buffer, pH 4.5 was mixed with the equilibrated resin in a 15 ml Falcon tube, and incubated at room temperature in a high speed rotating extractor (Taitec, Tokyo, Japan) at 50 rpm. The mixture was then transferred to a glass column and AaegOBP1 was eluted with citric acid buffer and analyzed by SDS-gel electrophoresis. The purest fractions were desalted on four 5-ml HiTrap desalting columns (GE Healthcare Life Sciences) in tandem by using water as mobile phase. Protein concentration was measured by the Quick Start Bradford Protein Assay (Bio-Rad, Hercules, CA).

### Fluorescence assays

Fluorescence measurements were done on a RF-5301 spectrofluorophotometer (Shimadzu, Kyoto, Japan) equipped with a magnetic stir bar. Samples in a 2-ml cell were excited at 337 nm, with the emission spectra recorded from 350 to 500 nm. Both emission and excitation slit were set a 5 nm. Data were recorded in high sensitivity, with automatic response time, fast scan speed, and sample pitch of 1 nm. AegOBP1 samples (10 µg/ml; ca. 0.7 µM, unless otherwise specified) were prepared in 100 mM ammonium acetate buffers. NPN titration were performed with acetate buffers pH 5.5 or pH 7. The other experiments, unless otherwise indicated, were done with acetate buffer pH 7. The fluorescence reporter and ligands were added by 0.5 or 1 µl aliquots of 1, 5, or 10 mM solutions in methanol. For displacement assays, 1 µl of 10 mM NPN (unless otherwise specified) was added, the solution was stirred in the cell for at least 10 min, stirring was ceased and spectra recorded. Then one aliquot of the test ligand was added, mixed for 2 min, and then the spectra were recorded. For NPN titration, the protein sample was stirred for 2 min, spectra recorded, 0.5 or 1 µl of 1 mM NPN solution was added and stirred for 2 min before recording. To avoid possible interferences, the light path was open only during recording and stirring was ceased at least 10 s before spectra were acquired.

 Data were analyzed with GraphPad Prism 6 (La Jolla, CA). For clarity, traces were reconstructed with GraphPad by transferring recorded data without normalization. To draw Figure 4, data were normalized (fluorescence recorded with AaegOBP1 and NPN, 100%) and for each concentration of the ligand mean ± SEM from three experiments were calculated in an Excel datasheet and transferred into Prism. Dissociation constants for NPN were determined by nonlinear regression curve fitting, one site and specific binding. MOP dissociation constant was calculated by measuring its competition for NPN binding. Thus, data were analyzed by nonlinear regression curve fitting (one site fits Ki), using the concentration of NPN (typically 5000 nM as HotNM) and Kd for NPN in nM (HotKdNM).

### Chemicals

NPN and DEET ( *N,N*-diethyl-3-methylbenzamide) were acquired from Sigma-Aldrich. MOP and PMD (p-mentan-3,8-diol) were gifts from Bedoukian Research, Inc. Picaridin (butan-2-yl 2-(2-hydroxyethyl)piperidine-1-carboxylate) and IR3535 (ethyl 3-[acetyl(butyl)amino]propanoate) were gifts from Dr. Kamal Chauhan (USA, ARS, Beltsville).

## Results and discussion

Update 1. Fluorescence reporter assay data with assessing binding of insect repellents to the yellow fever mosquito ( *Culex quinquefasciatus*) odorant binding protein AaegOBP1 Fluorescence reporter was *N*-phenyl-1-naphthylamine (NPN). Insect repellents used were DEET, PMD, Picaridin and IR3535. Mosquito oviposition pheromone was used as a positive control. Please see ReadMe file for details regarding each file. Please see the associated article for methods. The raw data for Figure 5C has been added in this version.

### Binding assays with insect repellents

**Figure 2.**
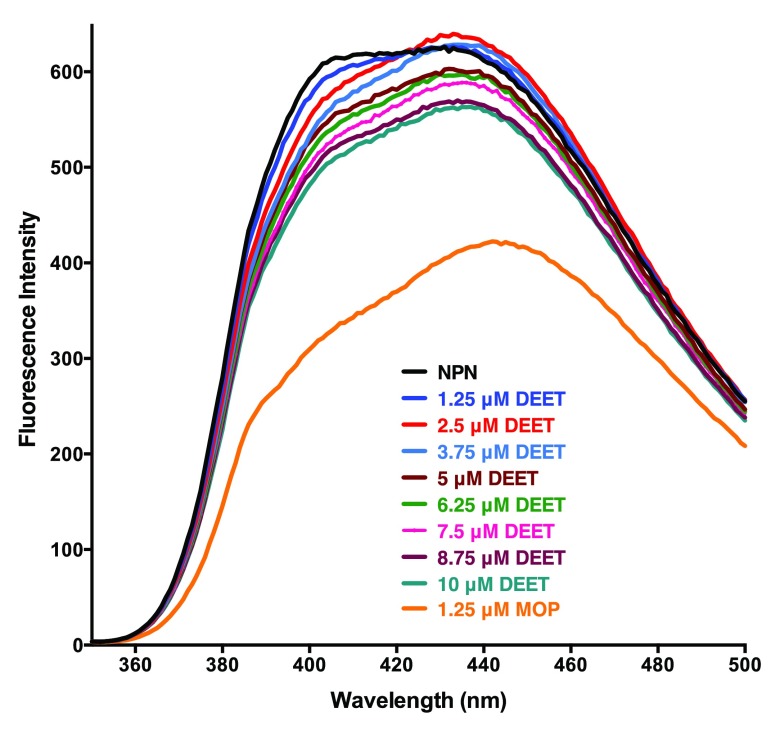
NPN fluorescence emission spectra. NPN bound to AaegOBP1 was excited at 337 nm and its emission spectra (black trace) was recorded. Then, increasing doses of DEET were added and finally one aliquot of MOP was added.

In preparation for binding assays of AaegOBP1 with insect repellents, we first measured the dissociation constant, Kd, for NPN: 3.31 ± 0.48 μM (n = 3). Subsequently, we measured fluorescence quenching by adding aliquots of insect repellents to a protein solution pre-equilibrated with 5 μM of NPN. To minimize solvent effect and reduce experimental error, we added 0.5 μl of 5 mM solutions of test ligands using a 2 μl pipette. As a positive control, we used a racemic solution of the mosquito oviposition pheromone (5 *R*,6 *S*)-6-acetoxy-5-hexadecanolide (MOP) ^37^ ( Figure 1), which has been previously demonstrated with the cold binding assay to bind to AaegOBP1 with apparently high affinity ^19^. Titration with DEET showed minor reduction in fluorescence intensity ( Figure 2) thus suggesting weak binding. By contrast, addition of 1.25 μM MOP led to almost one-third reduction in fluorescence intensity. Titration with other commercially available insect repellents, namely, picaridin, IR3535, and PMD gave similar results as DEET. Although our results suggest that all four repellents bound to AaegOBP1, it seems their affinities were too low to accurately measure dissociation constants. To complete the project and allow the high school investigator to measure at least one dissociation constant, we titrated MOP and this experiment led to unexpected and interesting results.

### Evidence for micelle formation

**Figure 3.**
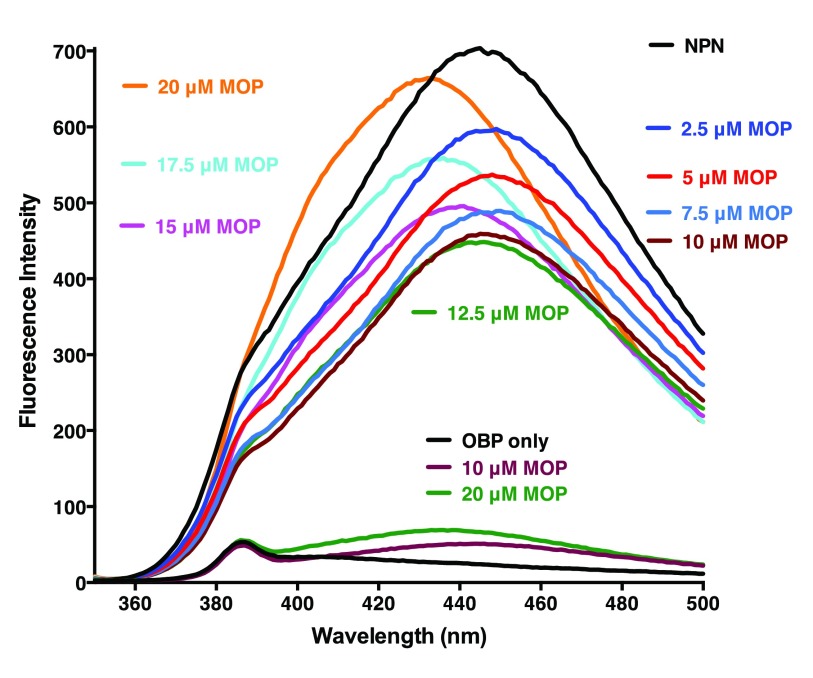
Binding of MOP to AaegOBP1. Following addition of NPN, fluorescence emission spectra were recorded with increasing doses of MOP. Note the decrease in fluorescence intensity (quenching) as the doses increases up to 10 µM and an increase in fluorescence and blue shift at higher doses. In a separate experiment, included in the lower part of the figure for comparison, fluorescence emission spectra were recorded with AgamOBP1 alone and after addition of MOP, but in the absence of NPN.

**Figure 4.**
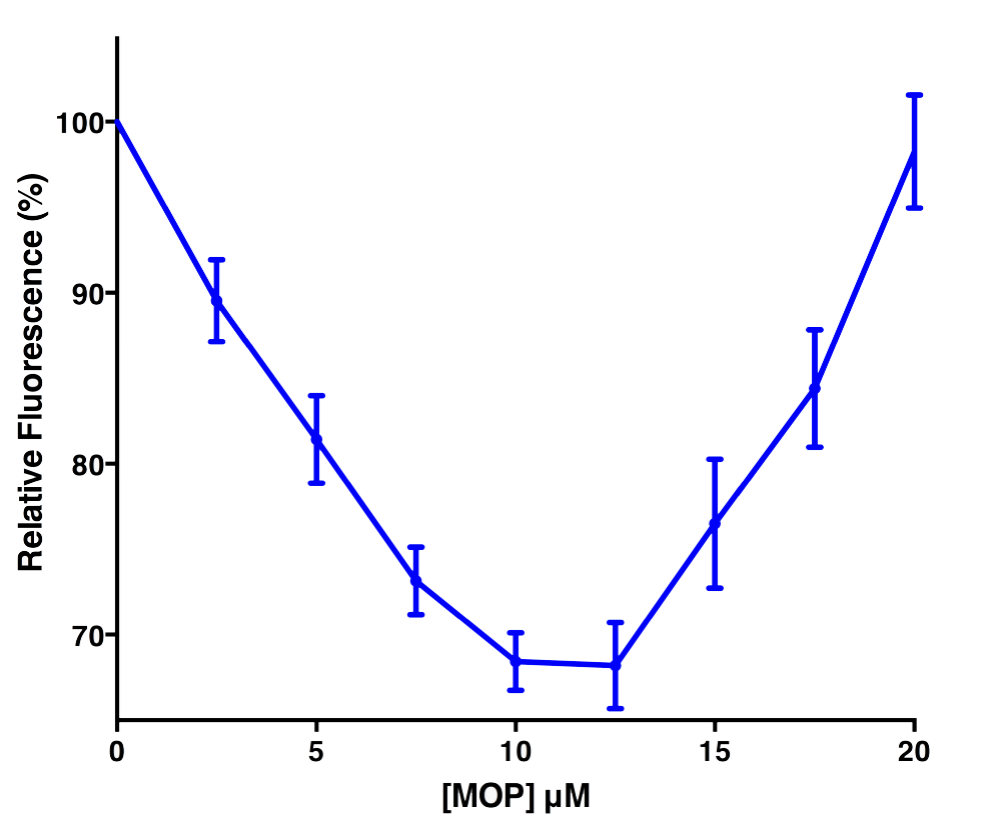
Effect of MOP on fluorescence emission of NPN bound to AaegOBP1. Emission maxima were normalized to display mean ± SEM from three experiments. MOP dissociation constant was calculate for the decreasing phase (0–12.5 µM). Note the increase in fluorescence emission thereafter.

**Figure 5.**
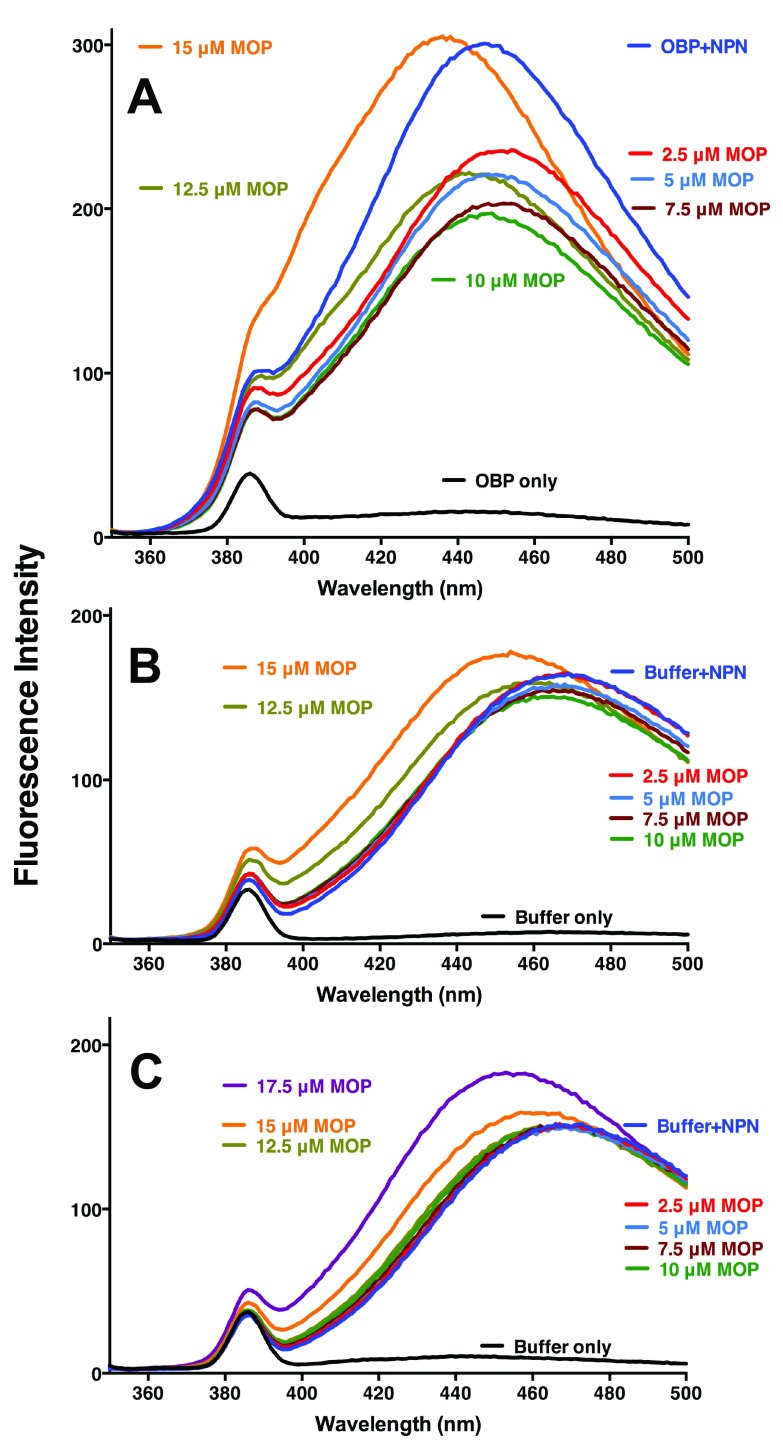
Titration of NPN fluorescence emission with MOP. ( **A**) NPN (2.5 µM) was added to a solution of AaegOBP1 (0.35 µM) in ammonium acetate buffer, pH 7. NPN (2.5 µM) was added to ammonium acetate buffer, ( **B**) pH 7 or ( **C**) pH 5.5.

Addition of MOP to solutions of AaegOBP1 pre-incubated with NPN caused a stepwise decrease in fluorescence intensity (2.5 μM to 10–12.5 μM doses), but rather than saturation further addition of MOP led to fluorescence increase and a blue shift. The senior investigator assumed it was an experimental error and repeated the experiments ( Figure 3). Quenching was observed when MOP was added up to 10–12.5 μM, but fluorescence increased thereafter and the maxima excitation wavelength shifted: AaegOBP1-NPN only, max 445 nm; AaegOBP1-NPN plus 2.5 μM MOP, 449 nm; AaegOBP1-NPN plus 20 μM MOP, 433 nm. Similar increase in fluorescence has been previously observed with high concentrations of ( *E*)-β-farnesene when titrating NPN fluorescence in the presence of an aphid OBP. Although unlikely, we tested in our case whether this unexpected fluorescence emission could be generated by MOP itself when bound to AaegOBP1 ^38^. The fluorescence emission levels generated even with AaegOBP1 plus 20 μM MOP (highest dose and no NPN) were indeed too low ( Figure 3) to explain the overall increase in fluorescence. We repeated these experiments and observed a clear U-shape curve with a minimum at 10–12.5 μM ( Figure 4). We measured the dissociation constant for MOP (2.64 ± 0.16 µM, n = 3) by considering only the first phase of the curve, i.e., by using the data generated by quenching or NPN replacement. Although the above experiments were conducted with reasonable low concentrations of ligands as compared to typical experiments ^29, 30^, we next examined the possibility of micelle formation with higher doses of MOP. We repeated titration of MOP using the same doses of the ligand, but reducing the concentrations of protein (0.35 µM) and fluorescence reporter (NPN, 2.5 µM) ( Figure 5). When added to ammonium acetate buffer at pH 7 ( Figure 5B) or AaegOBP1 in the same buffer ( Figure 5A), NPN fluoresced with emission maxima at 469 and 446 nm, respectively. Addition of MOP (2.5–10 µM) led to quenching of NPN in protein solution, but no significant change of NPN fluorescence in buffer solution. Addition of higher doses of MOP to a buffer solution, however, suggested the formation of micelles given the increase in fluorescence and blue shift observed at 12.5 and 15 µM of MOP at pH 7 ( Figure 5B) and at 15 and 17.5 µM at pH 5.5 ( Figure 5C), although we do not know the critical micelle concentration for MOP. The increase in fluorescence and blue shift were more pronounced in the presence of protein ( Figure 5A). It is, therefore, possible that the increase in fluorescence is a combination of micelle formation and other factor(s), which cannot be dissected by these experiments.

**Figure 6.**
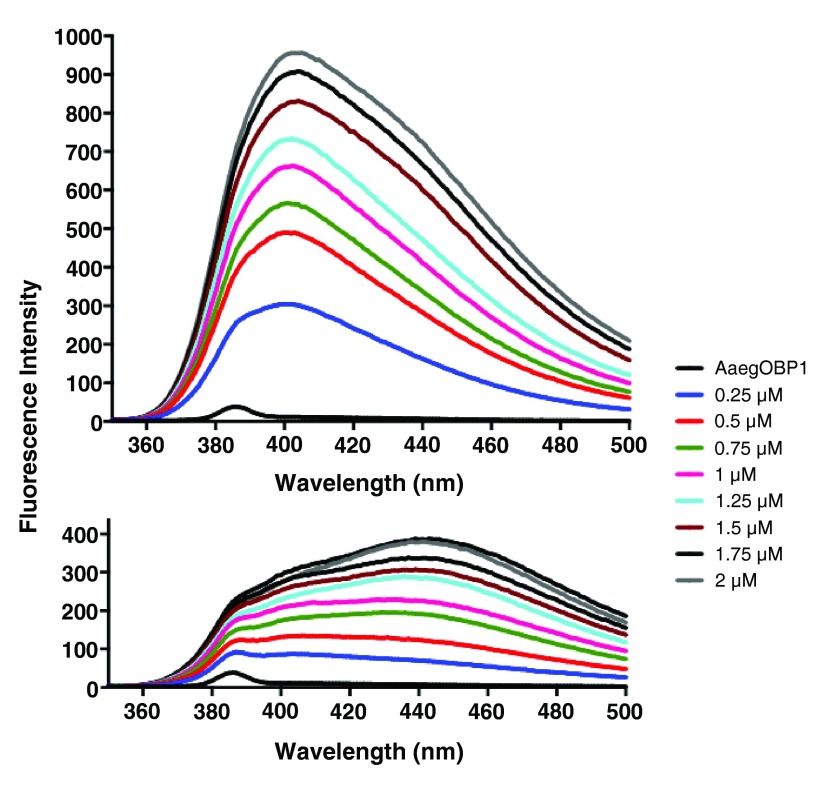
NPN fluorescence emission spectra obtained by titration at two pH values. Emission spectra at pH 5.5 (top traces) were considerably blue shifted relative to pH 7 (lower traces). Fluorescence intensity was also relatively higher at lower pH.

Lastly, we compared the fluorescence emission spectra obtained by titrating AaegOBP1 solutions at low and high pH values ( Figure 6). Interestingly, NPN showed a higher affinity for AaegOBP1 at pH 5.5 than at pH 7. Additionally, the emission spectra at low pH were blue shifted relative to pH 7 thus suggesting that at low pH NPN is accommodated in a more hydrophobic environment. It has been previously demonstrated that AaegOBP1 undergoes a pH-dependent conformational change. Although AaegOBP1 does not bind MOP at low pH, it has higher affinity for the fluorescence reporter: Kd = 1.07 ± 0.15 μM, pH 5.5; Kd = 3.31 ± 0.48 μM, pH 7. Lack of binding to odorants at low pH has been observed with the *Culex* orthologous protein, CquiOBP1 ^24^ and other OBPs, but insect fatty carriers bind ligands at low and high pH values ^39^.

## Conclusion

**Figure 7.**
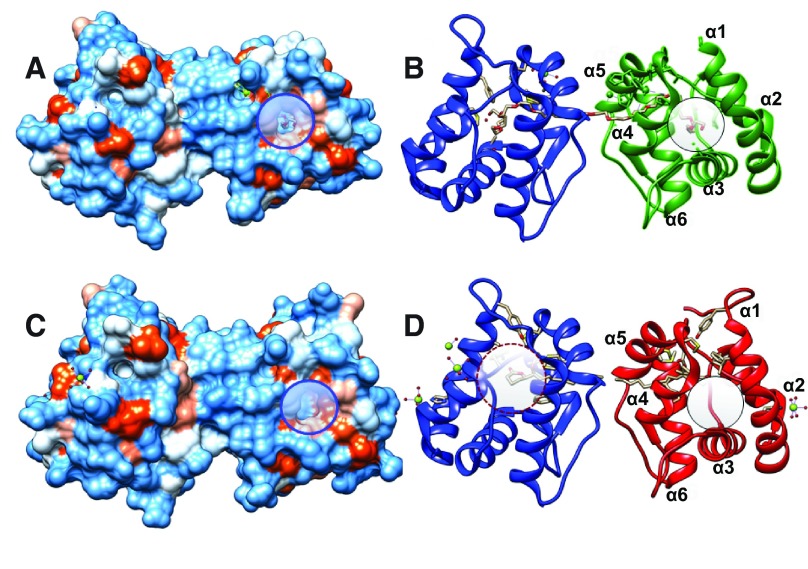
Structures of AaegOBP1 and CquiOBP1 bound to PEG and MOP, respectively. ( **A** and **C**) Hydrophobicity surfaces of AaegOBP1 and CquiOBP1. ( **B** and **D**) Ribbon displays of the same structures. A potential secondary binding site for MOP is highlighted with circles. It is occupied by PEG in AaegOBP1 but “empty” in CquiOBP1. The central cavity is highlighted in ( **D**) with a dashed circle and shows that only the polar head (lactone moiety) of MOP is housed in the core of the protein. Figure prepared with UCSF Chimera software.

A clear mechanistic explanation for the findings reported here must await further structural experimental data, particularly elucidation of crystal structures of AaegOBP1 bound to MOP and NPN separately as well as simultaneously. There are currently five structures of mosquito OBP1s deposited in Protein Data Bank (PDB), namely, AgamOBP1-PEG (PDB entry, 2ERB) ^21^ ( Figure 7A,B), AaegOBP1-PEG (3K1E) ^20^, CquiOBP1-MOP (NMR, 2L2C; crystal, 3OGN) ^19^ ( Figure 7C,D), AgamOBP1-DEET (3N7H) ^18^, AgamOBP1-sulcatone (4FQT) ^17^. Unfortunately, the only OBP-NPN complex (3S0B) ^40^ deposited in PDB is for an OBP from the European honey bee, AmelOBP14, which differs from classical OBPs for having two, instead of three, disulfide bridges. Here, NPN is bound in the central cavity of the protein. In CquiOBP1, MOP ( Figure 1) has its long lipid tail bound to a hydrophobic tunnel formed between helices 4 and 5 ( Figure 7D) and only its lactone/acetyl ester polar moiety is accommodated in part of the central cavity ( Figure 7D, dashed circle). It is, therefore, feasible that MOP and NPN were bound simultaneously, and given the vicinity between the two ligands MOP could cause quenching of NPN fluorescence. It has been shown that in AgamOBP1 DEET is localized at the edge of the binding pocket in the equivalent hydrophobic tunnel that accommodates the lipid tail of MOP in CquiOBP1 ( Figure 7D). Providing that NPN would bind in the central cavity, as in AmelOBP14, the distance between DEET and NPN would prevent quenching and, therefore, the “lack of binding” suggested by DEET titration ( Figure 2) might be interpreted with caution. If indeed mosquito OBPs have low affinity for DEET, it may explain, at least in part, the need to apply high doses of insect repellents. The unusual increase in fluorescence observed here might be explained at least in part by micelle formation. Unbound NPN, either displaced from AaegOBP1 or remaining in solution, could be housed in MOP-derived micelles and in this hydrophobic environment a blue shift and fluorescence increase are expected. It is also conceivable that at higher doses of MOP a second molecule of this ligand binds to AaegOBP1. There is another hydrophobic moiety bordered by helices α1 and α4 and occupied by PEG in the “apo-AgamOBP1”, which could possibly accommodate another ligand ( Figure 7, highlighted with circles). If so, NPN could be accommodated in a more hydrophobic environment thus causing a blue shift and additional increase in fluorescence. This change in NPN environment could be triggered by a conformational change. Of notice, NPN fluorescence emission was blue shifted at acidic pH (5.5) compared to neutral pH (7) ( Figure 6). Thus in the acidic conformation of AaegOBP1 NPN was more protected from the solvent, i.e., it is likely to be localized in a more hydrophobic environment. Previously, we have observed binding of two ligands to an insect OBP. The pheromone-binding protein from the silkworm moth, *Bombyx mori*, has been crystallized with two molecules of the bell pepper odorant, 2-isobutyl-3-methoxypyrazine ^41^. Likewise, fatty acid binding proteins have been demonstrated to bind two molecules of the same ligand, oleic acid ^42^. Recently, it has been suggested that DEET and NPN might bind simultaneously to AgamOBP1 ^17^, but experimental evidence showing increase in NPN fluorescence and blue shift data was missing. The hypotheses put forward here on the basis of our findings must await experimental evidence, in particular X-ray crystallography studies. Studies to test these hypotheses may lead to more effective fluorescence reporters and a better understanding of OBP odorant binding.

## Data availability

F1000Research: Dataset 1. Update 1. Fluorescence reporter assay data with assessing binding of insect repellents to the yellow fever mosquito ( *Culex quinquefasciatus*) odorant binding protein AaegOBP1, 10.5256/f1000research.5879.d41724 ^43^
