# Supplementary material for: Binding of a fluorescence reporter and a ligand to an odorant-binding protein of the yellow fever mosquito, Aedes aegypti
Source: F1000Res. 2015 Jan 9;3:305. Originally published 2014 Dec 12. [Version 2] doi: 10.12688/f1000research.5879.2 (PMC4309172; doi:10.12688/f1000research.5879.2)
Supplement: Peer review history

# Peer review: Referee response for version 2

**DOI:** 10.5256/f1000research.6433.r7334

**Published:** 2015-01-22

**Authors:** Kostas Iatrou, Spyros Zographos

**Affiliations:** 1 Institute of Biosciences and Applications, National Center for Scientific Research “Demokritos”, Athens, Greece; 2 Institute of Biology, Medicinal Chemistry & Biotechnology, National Hellenic Research Foundation, Athens, Greece

We are happy to see that our comments have been considered to be useful to the authors and appreciate the additional experimentation that was carried out in response to one of our comments. We do realize, of course, that molecular modeling by itself would not provide definitive answers to the working hypotheses put forward by the authors in relation to the binding pockets of AaegOBP1. Moreover, given that modeling is not an area of in-depth expertise for the specific group and that solid answers may eventually only come through X-ray crystallography, we feel that the authors have exhausted their capability to interpret fully their carefully documented observations. We would therefore encourage them to pursue a future investigation on this very interesting matter in collaboration with another relevant group. At the same time, we approve the indexing of the paper in its revised form.

We have read this submission. We believe that we have an appropriate level of expertise to confirm that it is of an acceptable scientific standard.

# Peer review: Referee response for version 1

**DOI:** 10.5256/f1000research.6281.r7176

**Published:** 2015-01-02

**Authors:** Paolo Pelosi

**Affiliations:** 1 Department of Chemistry and Agricultural Biotechnology, University of Pisa, Pisa, Italy

The phenomenon described in this paper is well known and documented in many papers. However, it has never been directly examined and explained in detailed. Therefore, it is nice and useful to have a focused study to describe and dissect such apparently anomalous behaviour once and for all.

I fully agree with the Authors that the formation of micelles is the most likely explanation. We have come across this same phenomenon several times and I have always discussed this fact with my students hypothesising the formation of micelles as the most likely reason behind this. A brief explanation of some anomalous binding curves can also be found in some of our published papers, most recently in Sun *et al. *(2012).

When a ligand capable of forming micelles also has affinity for the protein, we observe a decrease of fluorescence, followed by an increase when titrating the protein (the U curve observed in this paper). When the ligand has poor affinity for the protein, we only observe a constant increase in fluorescence. Sometimes we have also recorded a complex behaviour: the intensity of fluorescence experiences an increase at low concentration values of the ligand, then drops when more ligand is added. In this case, the phenomenon could be explained by assuming that the ligand enters the binding pocket without displacing the fluorescent probe. As the Authors point out such facts can occur and have been documented with OBPs and CSPs. The increase of fluorescence in such case would be the result of the increased hydrophobicity of the binding pocket due to the presence of a ligand, usually a highly hydrophobic molecule, as in the case of many pheromones of Lepidoptera and Diptera. As the concentration of the ligand increases, competition with bound 1-NPN can take over producing a decrease in fluorescence.

This study could be complemented (but not necessarily) by monitoring the intrinsic fluorescence of the tryptophan, which appropriately is located inside the binding pocket of the protein. Particularly in the case of DEET, which is an aromatic compound, if the molecule binds to OBP without being able to displace 1-NPN, we should observe a strong quenching of the tryptophan fluorescence.

Overall the paper is well written and the observed phenomenon clearly described and explained.

I have only one minor concern:

The Authors report the emission spectrum of 1-NPN alone (in Tris buffer at pH 7.4) with a maximum around 470 nm and in the presence of protein at  about 440. In my experience, I found that 1-NPN in buffer produces a peak with a maximum at 480, which is shifted in the presence of a binding protein to values generally between 400 and 410 nm, in some cases even below 400. This has been observed with a large number of OBPs, including some of mosquitoes, although not with the specific OBP used in this study. I suggest that the Authors double check these data, also in relationship to the instrument calibration.

I have read this submission. I believe that I have an appropriate level of expertise to confirm that it is of an acceptable scientific standard.

# Response

**Published:** 2015-01-02

**Authors:** Walter Leal

**Affiliations:** UC Davis, USA

We appreciate your time and effort to evaluate our article, and are delighted to hear that it meets your approval. We will certainly cite Sun *et al.* (2012) in the revised version of the article. The apparent formation of (E)-ß-farnesene micelle, as shown in your Figure 5, skipped our attention, perhaps because this phenomenon has never been examined and explained in detail. We will certainly give the appropriate credit in the revised version. Regarding your minor concern, we have verified wavelength accuracy per vendor’s instruction manual. We are, therefore, confident that the data set reported is accurate. It is worth pointing out, however, that apparent discrepancies may be explained at least in part by the proteins studied. For AaegOBP1, the NPN peaks at pH values 7 and 5 are quite different (our Figure 6), but in the presence of CquiOBP1 (Figure 5 in Leal *et al,* 2008) or AfunOBP1 (Figure 9, Xu *et al.*, 2010) the wavelength for the peaks at high and low pH are nearly the same, i.e., ca. 400 nm. All these studies were performed in our laboratory with the same instrument. Again, thank you very much for your suggestions. Sincerely, WSL & GML

# Peer review: Referee response for version 1

**DOI:** 10.5256/f1000research.6281.r7040

**Published:** 2014-12-29

**Authors:** Kostas Iatrou, Spyros Zographos

**Affiliations:** 1 Institute of Biosciences and Applications, National Center for Scientific Research “Demokritos”, Athens, Greece; 2 Institute of Biology, Medicinal Chemistry & Biotechnology, National Hellenic Research Foundation, Athens, Greece

This manuscript describes interesting results obtained in the course of what of an investigation initially designed as a high school project undertaken in order to deduce whether AaegOBP1, an odorant binding protein of the yellow fever mosquito *Aedes aegypti*, binds DEET and/or other known mosquito repellents such as icaridin, PMD and IR3535. The relevant experiments consisted of classical binding competition assays by the tested repellents against an AaegOBP1 pre-bound fluorescent reporter molecule, NPN, causing reductions in NPN-emitted fluorescent quenching with the latter serving as measure of mosquito repellent binding to AaegOBP1 resulting in displacement of the pre-bound NPN.

While the experiments suggested that the specific OBP may only bind the tested repellents with limited affinity relative to NPN, they also produced results that could not have been predicted *a priori*. The first concerned an unexpected property of a mosquito ( *Culex quiquefasciatus*) oviposition pheromone (MOP) that was used as positive control for binding to AaegOBP1. Thus, while titration AaegOBP1/NPN complexes by increasing quantities of MOP produced the anticipated reduction in NPN fluorescence, titrations with higher MOP doses led to gradual increases of fluorescence emitted by NPN accompanied by a wavelength shift toward the blue region of the spectrum. To explain this finding as well as the parallel observation that the same phenomenon also occurs at the same MOP concentrations in the absence of AaegOBP1, the authors have postulated the formation of MOP micelles forming a highly hydrophobic environment to which displaced and free NPN may bind.

The second intriguing finding has been that at a low pH of 5.5 at which AaegOBP1 is unable to bind MOP, this protein binds NPN with higher affinity relative to a neutral pH, causing higher emitted fluorescence with a concomitant blue-shift in the emission wavelength suggestive of the formation of a higher hydrophobicity environment to which NPN is bound. Based on these findings as well as the crystal structures of CquiOBP1 and *Anopheles gambiae* AgamOBP1, both AaegOBP1 orthologs, in complex with MOP and DEET, respectively, as well as the complex of the honey bee AmelOBP14 with NPN, the authors postulate the possibility that NPN and MOP could bind simultaneously to AaegOBP1 at a neutral pH. In turn, this possibility suggests that caution should also be exercised for the postulated conclusion regarding the low affinity binding of DEET to AaegOBP1, because DEET binding to a separate pocket might not necessarily result in displacement of NPN. 

Suggestions:

- For the first set of observations related to the postulated micelle formation by MOP at concentrations of 12.5 μM or higher, the hypothesized explanation is quite reasonable. A dynamic light scattering experiment using MOP in buffer alone could further strengthen the postulated hypothesis.  Moreover, a NPN titration experiment similar to that shown in Fig. 5B but at a pH 5.5, which should result in protonation e.g. of the acetoxy-group of MOP, could reveal whether an increase in micelle size occurs or not. This latter experiment could also provide additional suggestive evidence for the postulated creation of a more hydrophobic environment for NPN binding in AaegOBP1 at the acidic pH.

- For the structural considerations presented in the conclusions, as the authors indicate, co-crystalization of AaegOBP1 with NPN, MOP or both, will be required in order for conclusive interpretations to be drawn. Nevertheless, it is not clear to us why in a case of simultaneous binding of NPN and MOP (AaegOBP1-MOP-NPN complex), NPN should move to a different binding pocket of higher hydrophobicity producing higher fluorescence emission and a blue shift, only at higher MOP concentrations and not at lower ones. If, on the other hand, the requirement for higher MOP concentrations is interpreted as indicative of the formation of AaegOBP1-MOP-NPN-MOP complexes, a docking model should indicate whether enough space exists in the L-shaped tunnel of the AaegOBP1 monomer for simultaneous binding of 3 molecules.  

- For the low apparent affinity of AaegOBP1 for DEET, it is indeed possible that the binding of DEET and NPN to AaegOBP1 are not mutually exclusive, hence the low reduction in emitted NPN fluorescence in the presence of increasing concentrations of DEET. A docking model should indicate whether the possibility of nearby binding sites or even overlapping ones for NPN and DEET is predicted, which would lead to fluorescence quenching rather than reduction due to NPN displacement.

- Finally, the authors should provide a concluding statement as to whether and how these interesting findings relate to the contributions of OBPs in the mosquito's olfactory function under normal conditions.

We have read this submission. We believe that we have an appropriate level of expertise to confirm that it is of an acceptable scientific standard, however we have significant reservations, as outlined above.

# Response

**Published:** 2015-01-01

**Authors:** Walter Leal

**Affiliations:** UC Davis, USA

First of all, we would like to thank you for the time and effort to process and evaluate our article.  We were delighted to read the laudatory comments in your report. We have considered carefully your suggestions (thank you very much), and performed an additional experiment, which will be incorporated in Figure 5C. Specifically, we performed NPN titration with MOP in ammonium acetate buffer, pH 5.5. Although we agree that the suggested docking experiments (bullets 2 and 3) might add to the discussion, ultimately the structural hypotheses raised in the article shall be supported or refuted by X-ray crystallography-based structural evidence. While none of the authors is well versed in molecular modeling, we have the expertise and collaboration in place to rigorously test the structural hypotheses. In collaboration with our UCD colleague, Dr. David Wilson, Gabriel has been able to crystallize other OBPs, and we are confident that he/we will succeed in crystalizing AaegOBP1 complexes and address these questions. Obviously, time is uncertain in crystallography and the scope of the new work seems to belong to future publication(s). In the revised version of our *F1000Research *article, we will add comments regarding contributions of OBPs to mosquito olfaction, as suggested. Additionally, we will add results shown further evidence of micelle formation even at low pH, except that the effect was manifested at slightly higher concentrations of MOP. In sum, figures B and C are almost identical, but the blue shift and fluorescence increase were clearly observed starting at 15 and 17.5 uM at pH 5.5 (revised Fig. 5C) as compared to 12.5 and 15 uM (Fig. 5B). We do hope that now the article meets your approval.  Sincerely, WSL & GML
